# Investigating the Trend of the Great Slaty Woodpecker (*Mulleripicus pulverulentus*) Population Status in Western Nepal

**DOI:** 10.1002/ece3.71671

**Published:** 2025-06-27

**Authors:** Deelip Chand Thakuri, Hari Basnet, Laxman Prasad Poudyal, Prakash Chandra Aryal

**Affiliations:** ^1^ Bird Conservation Nepal (BCN) Kathmandu Nepal; ^2^ GoldenGate International College Kathmandu Nepal; ^3^ Nepalese Ornithological Union (NOU) Kathmandu Nepal; ^4^ Environment Protection and Study Center (ENPROSC) Kathmandu Nepal

## Abstract

The Great Slaty Woodpecker (*Mulleripicus pulverulentus*) has experienced a rapid population decline due to the loss of primary forest habitats across its range. Despite being classified as globally Vulnerable, detailed information regarding its status and distribution is largely insufficient and outdated. To address this, we conducted surveys from 2019 to 2021 in the western Terai Arc Landscape of Nepal, covering 29 transects, each 5 km long, to estimate the present population status, tree size of cavity trees, and overall distribution of the species in Nepal. We measured the diameter at breast height (DBH) within meter circular plots of 15 m radius at each woodpecker sighting location to explore the relationship between tree diameter and woodpecker presence. Additionally, we modelled the potential distribution of the habitat of Great Slaty Woodpecker across Nepal using available occurrence points. A total of 81 individuals were recorded across 14 transects, with 66 individuals within protected areas and 15 outside. Our findings demonstrated a direct correlation between tree DBH and woodpecker presence, indicating that large trees are critical for the species, with an average DBH of 61.26 cm for cavity trees where woodpeckers excavated cavities. Furthermore, we found that the total suitable habitat for the species in Nepal is approximately 6738 km^2^, with a significant portion located outside protected areas. The habitat in community forests and outside protected areas is particularly vulnerable to selective logging, posing a threat to the species. Therefore, further studies on the impact of logging on the Great Slaty Woodpecker are essential for effective conservation strategies.

## Graphical abstract

The study provides the potential habitat distribution of Great Slaty Woodpecker. Furthermore, the study examines the relationship between the occurrence of the species and tree size, specifically DBH.

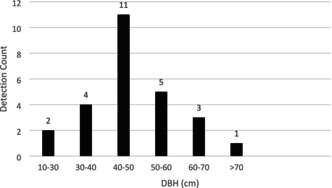

## 1 Introduction

Woodpeckers (Order Piciformes, Family Picidae) are a diverse group of birds found in various forest ecosystems worldwide, except in Australia and Antarctica, comprising 214 species (Gorman 2014). Renowned for their drilling abilities, they serve as key ecosystem engineers by excavating cavities in trees, which provide habitats for numerous cavity‐dwelling species, including birds (Nuthatches, Titmice, Owlets), mammals (Squirrels, Bats, Mice, Voles) and insects (Martin et al. 2021; Trzcinski et al. 2022; Baral 2023). Their cavity‐excavating behaviour not only supports biodiversity but also influences forest structure and dynamics, making them a vital component of a healthy forest ecosystem (Vergara and Schlatter 2004; Drever et al. 2008; Nappi et al. 2015). However, woodpecker populations are increasingly threatened by habitat loss due to deforestation, climate change, and other human‐induced factors, raising concerns about their conservation status globally (Short and Horne 1990; Lammertink 2014; Martin et al. 2021). Large‐bodied woodpeckers, in particular, are vulnerable to conventional forestry practices because they rely on large, mature trees for nesting, which are often the first to be removed during forest management (Short and Horne 1990). The decline of old‐growth forest has already led to the extinction of two of the world's largest woodpecker species, the Imperial Woodpecker (*Campephilus imperalis*) and the Ivory‐billed Woodpecker ( *Campephilus principalis* ), in the Americas (Short and Horne 1990).

**FIGURE 1:**
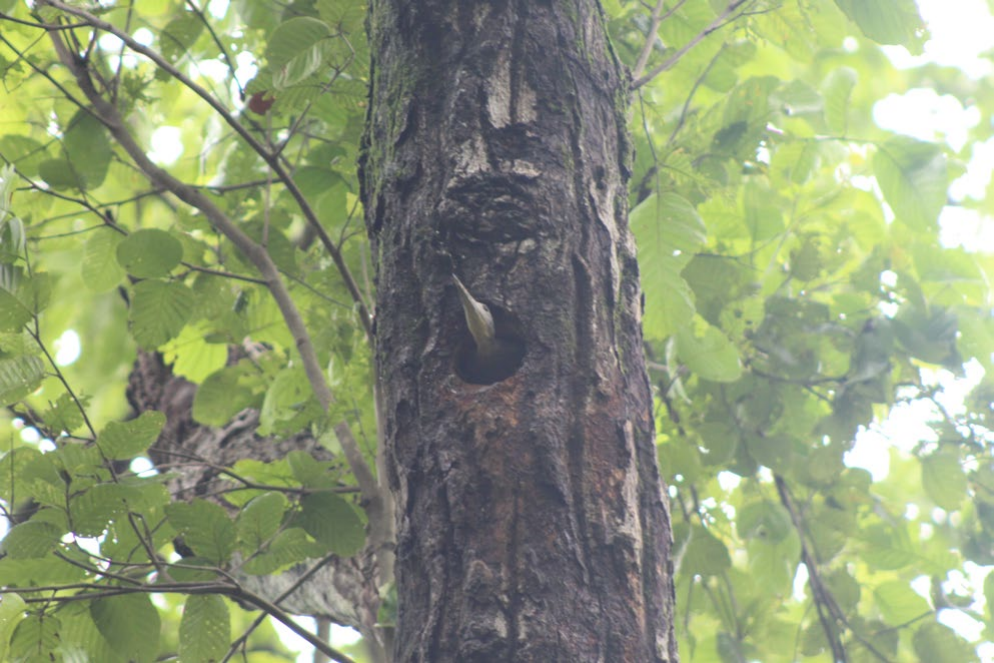
Great Slaty Woodpecker (female) in its cavity photographed in Bardia National Park, Nepal‐photographed (Photo‐ Deelip Chand Thakuri).

Among large‐bodied woodpeckers, the Great Slaty Woodpecker ( *Mulleripicus pulverulentus* ) (Figure 1) stands out as a prime example of a species that exemplifies the ecological importance and conservation challenges faced by woodpeckers (Lammertink et al. 2009; Lammertink 2014). As the largest woodpecker species found across 14 South and Southeast Asian countries, it plays a crucial role in its ecosystem, serving as both an indicator species and a facilitator of biodiversity through its cavity‐excavating behaviour (Lammertink 2014; Baral and Huettmann 2020). This species is primarily found in mature old‐growth forests, favouring climax Sal ( *Shorea robusta* ) and broadleaved and sub‐Himalayan moist deciduous forests in Nepal and India (Baral 2011; Kumar and Shahabuddin 2012; Inskipp et al. 2016; Joshi 2018). However, it is classified as “Vulnerable” on the IUCN Red List and “Endangered” in Nepal's National Red List, reflecting a concerning decline in its population due to habitat degradation and fragmentation (Inskipp et al. 2016; BirdLife International 2023). The Great Slaty Woodpecker's dependency on the integrity of forest ecosystems is evident as its nesting and foraging behaviour relies heavily on the presence of large trees and mature forest structures (Short and Horne 1990; Mikusinski 2006). The removal of large trees, often essential for its survival, poses a significant threat to the species' survival (Lammertink et al. 2009). Moreover, ongoing habitat degradation driven by logging and climate change has amplified the challenges faced by this species, leading to substantial population decline (Baral 2011; Inskipp et al. 2016; BirdLife International 2023).

Given this context, our study hypothesizes that the distribution and survival of the Great Slaty Woodpecker are strongly tied to forest integrity, particularly the availability of large trees and climatic conditions, both of which are sensitive to forest degradation and climate change. To explore this hypothesis, we address the following research questions: (1) How is the presence of the Great Slaty Woodpecker related to the availability of large trees in its habitat? and (2) How do climatic variables, such as temperature and precipitation, influence its habitat suitability in Nepal? By investigating these questions, this study aims to assess the present population status, evaluate the effects of climatic variables on habitat distribution, and examine the relationship between the Great Slaty Woodpecker's presence and the availability of large trees in the Western Terai Arc Landscape of Nepal. Ultimately, this study aims to provide critical insights into the ecological requirements of this threatened species, informing targeted conservation strategies to mitigate its decline.

## 2 Materials and Methods

### 2.1 Study Area

**FIGURE 2:**
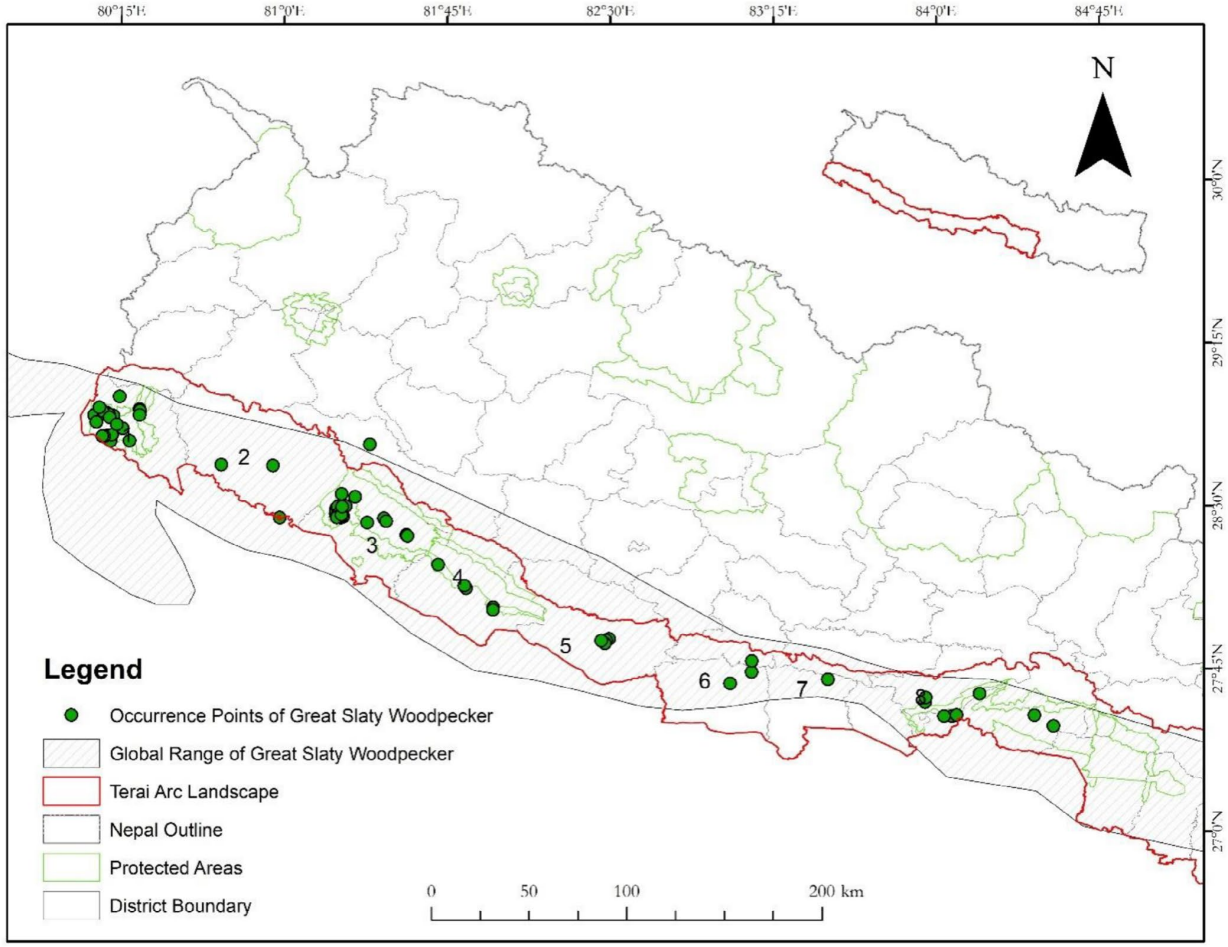
Study area with occurrence points of Great Slaty Woodpecker collected from the field and GBIF (1) Shuklaphanta National Park, (2) Kailali District, (3) Bardia National Park, (4) Banke National Park, (5) Dang District, (6) Kapilvastu District, (7) Arghakhachi District, and (8) Nawalparasi District.

The study was conducted in parts of the western Terai Arc Landscape (TAL), which extends from Nepal's Bagmati River in the east to India's Yamuna River in the west, connecting 16 protected areas across both countries. In Nepal, the TAL covers a vast area of 24,710 km^2^ and is home to a network of six protected areas, forests, agricultural lands, and wetlands, with over six million people depending on its forests for food, fuel, and medicine (WWF Nepal 2023). This study focused on three protected areas—Shuklaphanta National Park, Bardia National Park, and Banke National Park as well as mature forests in the foothills of Dang, Kapilvastu, Arghakhanchi, and Nawalpur districts (Figure 2).

The TAL is a vital repository for biodiversity and home to several endangered and charismatic mammals, including the Tiger ( *Panthera tigris* ), Asian Elephant ( *Elephas maximus* ), and Greater one‐horned Rhino ( *Rhinoceros unicornis* ) (Kanagaraj et al. 2011; Shrestha and Lapeyre 2018). Additionally, the region serves as a stronghold for woodpeckers in Nepal, hosting 15 of the 25 woodpecker species found in the country (Dahal et al. 2014; Baral and Huettmann 2020).

The landscape of the TAL is characterized by fine alluvium and clay‐rich swamps that support a diverse array of ecosystems, including tall grasslands, wetlands, and mixed deciduous forests dominated by Sal. However, large‐scale agricultural expansion and human settlement following malaria eradication over the past five decades have led to significant fragmentation and degradation of forest and grassland habitats in the region (Chanchani et al. 2014).

### 2.2 Data Collection

Potential habitats for the Great Slaty Woodpecker were identified using a combination of literature reviews, expert recommendations, and consultations with local birding groups. This approach ensured a robust and ecologically informed selection of study areas, as the species' occurrence is sparsely documented and heavily influenced by localized habitat conditions. Literature reviews provided baseline data on historical sightings and habitat preferences, while expert input helped prioritize regions with recent or persistent sightings. This methodology narrowed our survey focus to the TAL of Nepal, including protected areas (Shuklaphanta, Bardia, and Banke) and adjacent districts (Dang, Kapilvastu, Arghakhanchi and Nawalpur), where the species has been reliably reported (Inskipp et al. 2016).

Field data were collected through transect surveys, each 5 km long and 37 m wide on both sides. Surveys were conducted across seven blocks in protected and outside the protected areas (Table 1). Within the core areas of the protected areas, transects were strategically placed along the firebreak lines to mitigate risks associated with encounters with dangerous wildlife, such as tigers and elephants. Transects outside the protected areas were selected based on the presence of mature Sal forests, terrain features, and geographic accessibility.

**TABLE 1:** Transect with presence data. The table summarizes the number of transects surveyed, transects with species presence, and the total number of individuals counted in each area.

S.N.	Area	Transects surveyed	Transect with species	Individuals counted
1	Shuklaphanta National Park	9	5	35
2	Bardia National Park	5	3	19
3	Banke National Park	7	2	12
4	Dang	3	1	3
5	Kapilvastu	2	1	2
6	Arghakhanchi	1	1	5
7	Nawalparasi	2	1	5

Surveys were conducted during the breeding season (March–July) in 2019 and 2021. No surveys were conducted in 2020 due to COVID‐19 restrictions. Each transect was surveyed once, with surveys conducted from 0700 to 1200 h in the morning and opportunistic surveys conducted from 1500 to 1700 h in the evening. Three surveyors walked at a pace of 1 km per hour, recording observations of Great Slaty Woodpecker individuals sighted. The GPS location of the observation point (from where the bird was sighted) and the bird's location were recorded, and the perpendicular distance between these points was calculated using Google Earth Engine (Gorelick et al. 2017). If more than one bird was observed in two or more trees, the GPS point of the first sighted bird was recorded for habitat measurements, but all individuals were counted for density analysis. Additional observations made outside the designated transects and opportunistic sightings after 2021 were noted and utilized only for distribution modeling to better understand the species' overall distribution.

We compiled 156 GPS locations (occurrence points) of the Great Slaty Woodpecker collected between March 2019 and May 2024. These occurrence points were sourced from our field surveys (*N* = 21), opportunistic sightings (*N* = 5), and published records accessed through the Global Biodiversity Information Facility (GBIF) and defined as “occurrence points” which were used for habitat modeling. All GBIF records were rigorously validated by cross‐referencing original sources, consulting experts, and removing outliers such as occurrences outside the species' known range to ensure data reliability.

### 2.3 Habitat Data

At each detection point, where the species was observed (designated transects and opportunistic sightings before 2021), a circular plot of 15 m radius was established to measure the tree diameter at breast height (DBH), tree height, forest type and logging status, with trees lager than 10 cm DBH inside the plot assessed for these features. To determine the logging status, we observed the presence of tree stumps in the area, categorizing it as logged if stumps were observed. Additionally, we systematically searched tree cavities during the transect walks by visually scanning trees using binoculars (Olympus 8*42). When the tree cavities were identified, the DBH of the cavity tree, tree height, the number of cavities and the cavity height above the ground were recorded. Tree height and cavity height were measured using clinometer, while DBH was measured using a DBH tape. Great Slaty Woodpecker cavities were confirmed when any activity (going in and out) from the cavity was observed. The cavities where birds were not seen were checked several times (for three continuous days) by local assistant (nature guide) to confirm they belonged to the Great Slaty Woodpecker. All habitat data were collected after the completion of the transect survey to maintain survey efficiency and minimize disturbance to the birds.

For transects where the species was not observed, we established circular plots of 15 m radius at random points along the transect line to collect habitat data. These plots were treated as “absence data” for regression analysis, representing areas where the species was not detected during the survey period. The same habitat variables (DBH, tree height, forest type, and logging status) were measured in these absence plots to ensure comparability with presence data. Tree height refers to the vertical measurement from the base of a tree to its highest point. To determine this, we used a clinometer along with a measuring tape. First, the horizontal distance from the observer to the tree was measured. The clinometer was then used to measure the angle of elevation to the top of the tree. A 1.5‐m stick was held vertically at the base of the tree to serve as a reference point for taking the angle measurement. The height of the tree was calculated using the following formula:

$$\text{Tree height}\;\left(m\right)=\left(\text{Horizontal distance in meters}\times \tan\alpha \right)+1.5\;m$$

where *α* is the angle of elevation to the treetop. This approach ensured consistent tree height measurements throughout the study.

Cavity height was measured as the distance from the ground to the first cavity in the tree. We measured the cavity height using a clinometer and measuring tape, following the same method as for tree height.

Data analysis the density of Great Slaty Woodpecker was calculated using the formula:

$$\begin{array}{cc} \text{Density}\;\left(D\right)\;=\; & \text{Total number of individuals counted in the survey}\;\left(N\right)\\ & /\text{Total area surveyed}\;\left(A\right) \end{array}$$

where *N* = Total number of individuals counted (81) and *A* = (10.73 km^2^ from 29 transects: length 5 km × 0.074 km width). All these transects fell within the confirmed Great Slaty Woodpecker habitat (mature sal forests see Data Collection for selection criteria), ensuring ecological relevance. This yielded a density of 7.54 ± 0.72 individuals/km^2^.

To evaluate the relationship between the presence of the Great Slaty Woodpecker and tree DBH, two separate regression models were constructed: one using average stand DBH and another using maximum DBH as independent variables. The presence or absence of the species were treated as dependent variables (binary 1 for presence and 0 for absence).

A generalized linear model (GLM) with logistic regression framework was employed as the response variable was binary. The models were implemented using (R Core Team 2018). Model diagnostics, including checks for multicollinearity and residual analysis, were performed to ensure the robustness of the results. Statistical significance was assessed at *p* < 0.05.

This analysis aims to identify the suitable habitat and environmental factors influencing the distribution of the Great Slaty Woodpecker in Nepal. For this, we used MaxEnt, which is widely used for modeling species distributions using geo‐referenced occurrence data and environmental variables (Phillips et al. 2006). This tool selects background locations to contrast with presence locations, estimating the density of presences across the landscape (Phillips et al. 2004; Merow et al. 2013). This tool has been applied in wildlife habitat mapping and evaluating environmental impacts on species distribution (Mishra et al. 2018; Thapa et al. 2018; Zhang et al. 2019; Suwal et al. 2020).

We used MaxEnt (version 3.4.1) with 11 selected bioclimatic variables (Figure 4) and occurrence points to model predicted habitat distributions for the Great Slaty Woodpecker in Nepal. The bioclimatic variables were downloaded from WorldClim (version 2.1), a global climate database available at a spatial resolution of 30 arc‐seconds (1 km^2^). All selected variables were included after confirming acceptable multicollinearity levels (|*r*| < 0.70) (Dormann et al. 2013). Occurrence data were thinned and spaced at least 1 km apart to minimize spatial autocorrelation. Model settings included 1000 maximum iterations and 10 replicates. Model performance was evaluated using threshold‐independent and threshold‐dependent methods. AUC values below 0.7 indicate poor performance, 0.7–0.9 suggest moderate performance, and values above 0.9 denote excellent performance (Pearce and Ferrier 2000). True Skill Statistics (TSS), calculated as Sensitivity + Specificity − 1, assessed threshold‐dependent performance, with values ranging from −1 to 1 (Allouche et al. 2006). We averaged the TSS scores from all 10 model runs using R software (R Core Team 2018). Additionally, the habitat map was clipped by land use to identify the current habitat status across various land use forms in Nepal.

## 3 Results

### 3.1 Distribution and Status

We recorded 81 individuals of the Great Slaty Woodpecker from 14 out of 29 surveyed transects (Table 1) with sightings documented at 21 locations ranging from 168 to 532 m above sea level. Among these locations, 16 sightings were within protected areas, while five were outside. The flock sizes ranged from a single individual to eight individuals, with an overall density of 7.54 ± 0.72 individuals/km^2^. Areas with no logging had slightly higher density of birds (7.70 ± 0.97 individuals/km^2^) compared to areas with logging (7.20 ± 1.00 individuals/km^2^).

Inside protected areas, we recorded a total of 66 individuals, with a density of 8.49 ± 0.96 individuals/km^2^, while 15 individuals were recorded outside the protected areas, with a density of 5.06 ± 0.78 individuals/km^2^ (Table 2). The highest number of individuals was recorded in Shuklaphanta National Park (35), followed by Bardia National Park (19) (Table 1). Notably, using opportunistic surveys, we detected the Great Slaty Woodpecker for the first time in the Arghakhanchi and Rupandehi (Opportunistic) districts.

**TABLE 2:** Table showing the results of transect surveys.

Survey areas	Number of transects surveyed	Total individuals	Density (individuals/km^2^)	Standard error (SE)
Overall	29	81	7.54	0.72
Inside protected areas	21	66	8.49	0.96
Outside protected areas	8	15	5.06	0.78
No logging	20	57	7.70	0.97
Logging	9	24	7.20	1.00

### 3.2 Tree Selection and Characteristics of Cavities

We recorded 19 trees with cavities of Great Slaty Woodpecker, the majority of cavities (17) found in sal and one cavity each was found in harro ( *Terminalia chebula* ) and sajh (*Terminalia elliptica*) trees. The mean height of these cavity trees was 27.89 ± 2.72 m, with an average DBH of 61.26 ± 24.75 cm and an average of 3.78 ± 2.04 cavities per tree. The average height of the cavities from the ground was 19.47 ± 3.13 m (Table 3).

**TABLE 3:** Description of cavity tree.

S.N.	Variable	Height (m)	DBH (cm)	No. of cavities	Height of first cavity from the ground (m)
1	Mean	27.89	61.26	3.78	19.47
2	SD	2.72	24.75	2.04	3.13
3	Minimum	24.00	41.00	1.00	13.00
4	Maximum	33.00	156.00	8.00	25.00

The probability of occurrence of the Great Slaty Woodpecker showed a significant positive association with both average stand DBH and maximum DBH. The slope coefficient for average stand DBH was 0.026 (*p* < 0.001), suggesting that for every unit increase in average stand DBH, within the measured values, the likelihood of the Great Slaty Woodpecker's presence increases significantly. Similarly, the slope coefficient for the largest DBH tree was 0.024 (*p* < 0.001), demonstrating that for every unit increase in maximum DBH, the likelihood of the Great Slaty Woodpecker's presence also increases significantly (Table 4).

**TABLE 4:** Probability of occurrence of the Great Slaty Woodpecker with DBH.

Model	Coefficient	Estimate	Standard error	*t*‐value	*p*
Average stand DBH	Intercept	−0.358	0.251	−1.427	0.161
	Average DBH	0.026	0.006	4.056	0.0002
Maximum DBH	Intercept	−0.65	−0.651	−1.999	0.0528
	Maximum DBH	0.024	0.005	3.998	0.0002

**FIGURE 3:**
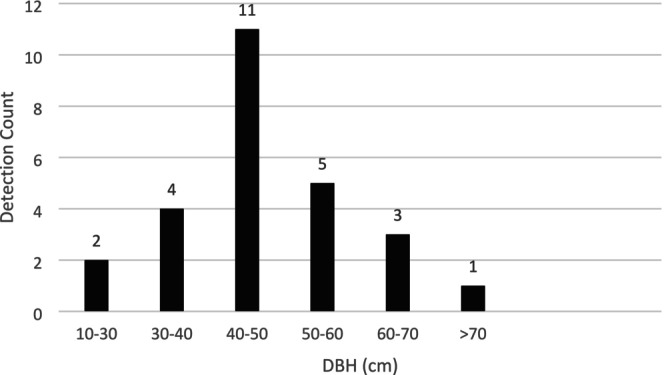
Detection frequency of the Great Slaty Woodpecker across different diameter at breast height (DBH) classes.

Furthermore, the detection frequency of the Great Slaty Woodpecker varied across different DBH classes, with the highest occurrence in trees with a DBH of 40–50 cm (*n* = 11 detections), followed by 50–60 cm (*n* = 5) and 30–40 cm (*n* = 4). Detection decreased in both smaller and larger DBH classes, indicating a preference for mid‐sized trees (Figure 3).

### 3.3 Potential Habitat Distribution

**FIGURE 4:**
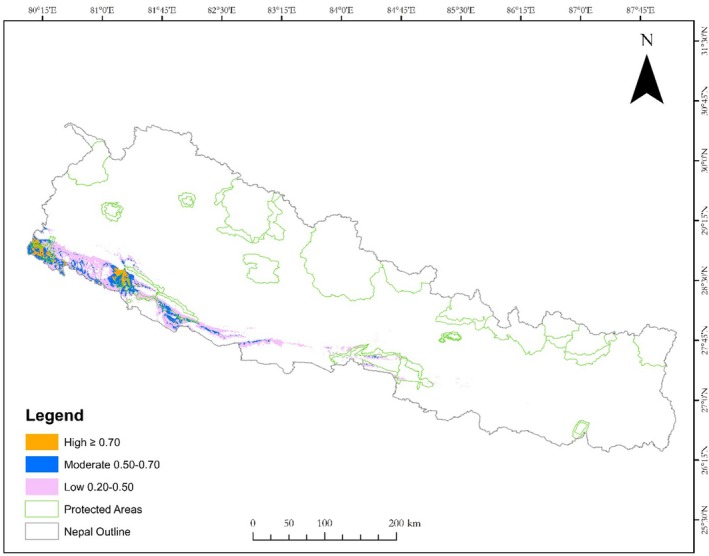
Habitat suitability distribution of the Great Slaty Woodpecker.

The potential suitable habitat for the Great Slaty Woodpecker is predominantly located in Far and Mid‐western Nepal, with a gradual decline toward Western and Central Nepal (Figure 4). A total of 6738 km^2^, representing 4.33% of Nepal's total area, has been identified as potential suitable habitat for the species (Figure 4). Approximately 32.38% (2181.88 km^2^) of this habitat is within existing protected areas, while the remaining habitat is outside these areas (Table 5). Bardia National Park and its buffer zone encompass the largest proportion, containing 16.26% of the total suitable habitat, whereas Parsa holds the smallest share at 0.78%. In terms of highly suitable habitats, Shuklaphanta National Park accounts for 37.5%, Bardia National Park for 23.7%, and the remaining 38.5% lies outside protected areas (Table 5).

**TABLE 5:** Habitat suitability areas for Great Slaty Woodpecker within and outside protected areas in Nepal.

Suitability	ShNP (km^2^)	BNP (km^2^)	Banke (km^2^)	CNP (km^2^)	PNP (km^2^)	Protected areas (km^2^)	Outside protected areas (km^2^)	Total area (km^2^)
High (≥ 0.75)	238.69	151.09	0.8	0	0	391	245.5	636.5
Medium (0.50–0.70)	287.93	384.14	50.186	21.828	0	744.084	1088	1832.084
Low (0.27–0.50)	106.967	560.59	81.65	245.29	52.3	1046.797	3223	4269.797

Abbreviations: BNP, Bardia National Park; CNP, Chitwan National Park; PNP, Parsa National Park; ShNP, Shuklaphanta National Park.

### 3.4 Important Bioclimatic Variables and Land Use

**FIGURE 5:**
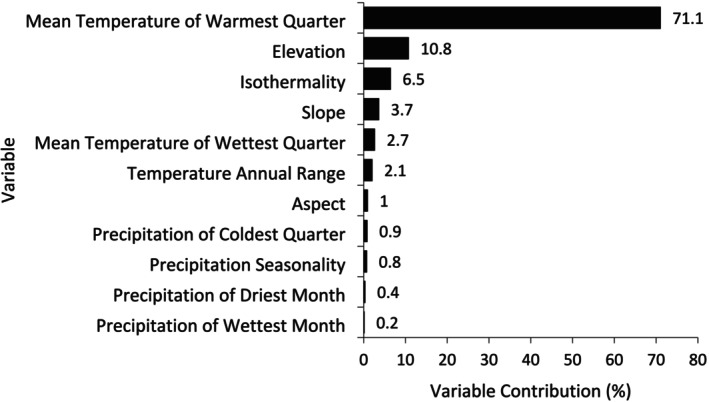
Variable contribution for the species distribution model.

**FIGURE 6:**
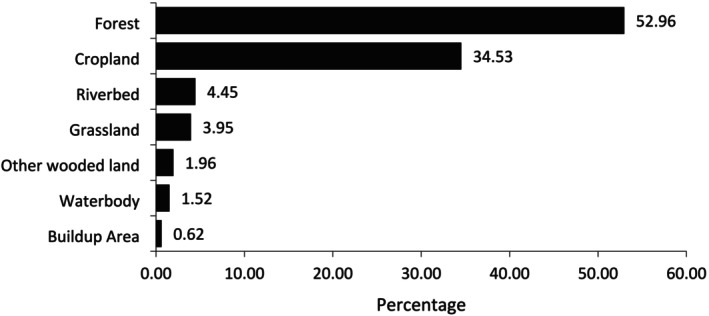
Habitat distribution of the Great Slaty Woodpecker according to land use.

Of the 11 variables analyzed, Mean Temperature of the Warmest Quarter (BIO 10), elevation, and Isothermality (BIO 3) were identified as the most important variables determining habitat suitability, while Precipitation Seasonality (BIO 15), Precipitation of the Driest Month (BIO 14), and Precipitation of the Wettest Month (BIO 13) were the least important (Figure 5). The most suitable habitat (52.96%, or 3556.10 km^2^) was found in forested areas, followed by cropland and riverbeds. The least suitable habitat (0.62%, or 41.94 km^2^) was found in built‐up areas (Figure 6).

### 3.5 Model Accuracy

The model accuracies were relatively high, with an AUC of 0.96 and a TSS of 0.85 (Appendix I). A threshold of 0.08 was established to maximize the sum of sensitivity and specificity. This threshold was used to calculate TSS and to convert the continuous habitat suitability map into a binary suitable/unsuitable map.

## 4 Discussion

### 4.1 Distribution and Status

Our findings provide crucial insights into the distribution and status of the species within the TAL of Nepal. The species was recorded in 14 out of 29 surveyed transects, indicating its widespread but patchy presence in the TAL of Nepal. The documentation of the Great Slaty Woodpecker in new locations, such as the Arghakhanchi and Rupandehi districts, along with earlier records in Dang (Thakuri and Nepal 2009; Inskipp et al. 2016; Khanal and Dangi 2020; Khanal et al. 2024) and Kapilvastu districts (Pandey and Ghimire 2018), underscores the significance of areas beyond the protected area system for this species. These regions, located between Chitwan National Park in the central lowlands and Banke National Park in the mid‐western lowlands, likely serve as fragmented corridors connecting isolated populations. Conserving these forested areas is crucial for facilitating gene flow, dispersal, and movement of individuals, which are essential for maintaining a healthy and resilient population across a broader landscape (Baral 2011; Brás et al. 2013; Inskipp et al. 2016).

We recorded 81 individuals across the surveyed transects, representing nearly half of the estimated national population of the Great Slaty Woodpecker in Nepal (Inskipp et al. 2016). This suggests that the actual population may exceed earlier estimates of 190 to 250 individuals, which were based on expert opinions rather than systematic surveys (Baral 2011; Inskipp et al. 2016). The highest number of individuals was recorded in Shuklaphanta National Park, followed by Bardia National Park. However, this contrasts with a previous study conducted that reported higher numbers in Bardia National Park (Baral 2011). These contrasting results may be attributed to variations in survey intensity and area coverage, as we conducted the survey in nine transects in Shuklaphanta compared to five in Bardia. Both protected areas are, however, critical strongholds for the species, as evidenced by regular sightings in these areas (Ebird 2021).

Our findings also support the notion that Banke National Park holds a considerable population of the Great Slaty Woodpecker, being a less explored area compared to Shuklaphanta and Bardia. In Banke National Park, we counted a total of 12 individuals of the Great Slaty Woodpecker in nine of the surveyed transects. Likewise, Baral (2011) suggested that Banke could be another suitable habitat for the species, as the area was understudied. The presence of the Great Slaty Woodpecker in these areas highlights the need for regular monitoring and exploration of understudied areas to understand the species' distribution and population status.

### 4.2 Tree Selection and Characteristics of Cavities

The occurrence of the Great Slaty Woodpecker is strongly associated with large trees, particularly those with DBH exceeding 30 cm (Lammertink et al. 2009). Likewise, our result also demonstrated a clear preference for trees ranging from 30 to 70 cm in DBH (Figure 3). Furthermore, our regression analysis revealed a significant relationship between the presence of the Great Slaty Woodpecker and both average stand DBH and maximum DBH. The average stand DBH model showed that for every unit increase in DBH, the likelihood of the species increased significantly. Similarly, the maximum DBH model indicated a significant positive effect, with the probability of occurrence rising markedly when the maximum DBH of the stand exceeded 40 cm (Table 4). These findings align with previous studies that documented the species' preference for large trees, which provide stable substrates for cavity excavation and foraging opportunities (Lammertink 2004; Kumar and Shahabuddin 2012). However, the strong preference for large trees makes the Great Slaty Woodpecker particularly vulnerable to logging and forest degradation, as these activities often target the largest and most commercially valuable trees (Lammertink et al. 2009; Lammertink 2014; Baral and Huettmann 2020).

Our findings suggest that the Great Slaty Woodpecker prefers to excavate cavities in larger trees, with an average DBH of 61 cm, which is slightly higher than the 53.5 cm reported in India (Kumar and Shahabuddin 2012). This variation suggests that the species may exhibit some regional variation in habitat preferences, possibly influenced by local forest structure, ecological factors or the availability of tree sizes. Nonetheless, this preference emphasizes the species' dependence on mature forests with substantial tree sizes (Lammertink 2014; Inskipp et al. 2016; BirdLife International 2023). Although our study did not specifically assess the impacts of logging, we observed a fewer individual in areas affected by logging (Appendix II). This pattern is consistent with previous findings where the Great Slaty Woodpecker was less abundant in logged forests in Myanmar and Indonesia, highlighting a negative correlation between the species' presence and logging disturbances (Lammertink et al. 2009). In Malaysia, no sightings of the Great Slaty Woodpecker were reported in logged forests (Styring and Ickes 2001), further underscoring the negative effects of logging on this species.

Our study primarily focused on the detection of the Great Slaty Woodpecker and its cavity trees in relation to tree size. Further research is necessary to gain a more comprehensive understanding of its nesting ecology. Future research of specific characteristics of cavities excavated by the Great Slaty Woodpecker and how these preferences influence its habitat selection and breeding success can shed light on species ecology, specifically micro‐habitat selection and behavior. Such insights are crucial for developing targeted conservation strategies to mitigate the ongoing threats to the Great Slaty Woodpecker and its habitat.

### 4.3 Potential Habitat Distribution

The MaxEnt model employed in our study exhibited a high discriminative ability (AUC = 0.967), effectively identifying suitable habitats for the Great Slaty Woodpecker in Nepal. This high AUC value indicates the model's strong capacity to differentiate between suitable and unsuitable habitat (Phillips et al. 2004). The strong influence of mean temperature of the warmest quarter (MTWQ) on the Great Slaty Woodpecker's distribution highlights its reliance on thermally stable microclimates in tropical sal forests. The species' preference for areas with moderate temperature fluctuations (indicated by Isothermality) suggests that it may be less adaptable to extreme climatic variability, which is expected to increase under climate change (Williams et al. 2021). Rising temperatures during the warmest quarter could alter food availability and increase physiological stress, further threatening the species (Quratulann et al. 2021; Rajpar et al. 2022). As a cavity‐nesting species, it likely avoids extreme heat stress during breeding and roosting, which could desiccate nests or reduce foraging efficiency (Wiebe 2001; Scheffers et al. 2014). High MTWQ may also correlate with sal forest health, as sal thrives in specific thermal regimes; temperature extremes could degrade tree quality, limiting cavity availability (Chitale and Behera 2012). This thermal dependency underscores vulnerability to climate change—rising temperatures may contract suitable habitat, and foraging urging conservation prioritization of thermally buffered forest stands to ensure long‐term viability (Parmesan et al. 2003; Thomas et al. 2004; Styring et al. 2016). However, we observed that rainfall had the lowest contribution to the Great Slaty Woodpecker's distribution. This is likely due to low precipitation levels in the western part of Nepal (Timilsina et al. 2007), resulting in minimal variation in moisture availability across the landscape. Additionally, the habitat is dominated by sal trees, a semi‐deciduous species (Pandey and Shukla 2001), that thrives in well‐drained soils across a range of moisture conditions (Dinerstein 1979; Gautam and Devoe 2006). The region's low groundwater table and porous soils further prevent prolonged surface inundation, even during the monsoon season (Bolton 1976). Given these factors, rainfall does not appear to significantly influence the distribution of key habitat features, explaining its minimal contribution to the habitat suitability model.

The analysis identified the mid‐western and far‐western lowland forested areas, particularly within Shuklaphanta and Bardia National Parks, as a suitable habitat for Great Slaty Woodpecker (Figure 4). Field surveys supported this finding, with the species detected in 8 out of 14 transects (57%) surveyed across the protected areas, accounting for 67% of all recorded individuals. These results align with evidence demonstrating the efficacy of protected areas in mitigating anthropogenic threats to biodiversity (Araújo et al. 2011; Alagador et al. 2014). However, while conservation efforts predominantly target protected areas, species outside these boundaries remain disproportionately vulnerable to habitat degradation (Gray et al. 2016; Boakes et al. 2019). Our habitat suitability modeling revealed that as much as two‐thirds of the Great Slaty Woodpecker habitats (67.62%) lie outside protected areas, where it is exposed to various threats, including selective logging in community forests (Shrestha 2008; Oli and Subedi 2015). These findings emphasize the need for conservation strategies that extend beyond protected areas, as species outside these zones remain highly vulnerable. Such approaches are vital for addressing the species' reliance on unprotected forests and mitigating regional extinction risks (Gray et al. 2016; Boakes et al. 2019).

Furthermore, our model also predicted substantial habitat of the Great Slaty Woodpecker in less‐explored areas such as Banke, Dang and, the corridors between Bardia National Park and Shuklaphanta National Park including the Basanta and Khata corridors (Figure 4). Although areas lack formal protection, evidence of species presence has been documented (Inskipp et al. 2016; UNDP 2019; Chaudhary 2023). These areas outside protected areas are crucial for maintaining connectivity for isolated population. As the population in these areas can face extirpation (Boakes et al. 2019). Without targeted conservation efforts, the Great Slaty Woodpecker could face extirpation from these regions, as evidenced by population declines of up to 90% in areas affected by deforestation (Lammertink et al. 2009; Kumar et al. 2011).

### 4.4 Habitat Distribution by Land Use

Evidence suggests that deforestation of mature forests has caused a population decline of up to 90% in Great Slaty Woodpecker (Lammertink et al. 2009). Additionally, (Kumar et al. 2011) did not record any individuals of the species in the managed Sal forests in India, indicating the species' dependency on large and mature trees. Our model indicated that 53% of predicted suitable habitat lies in the forested areas that are currently threatened by excessive forest fires, overgrazing, linear infrastructure development, and selective logging (Shrestha et al. 2010; Chaudhary et al. 2016). These disturbances degrade the state of mature trees, which are essential for the foraging and nesting of large‐bodied woodpeckers like the Great Slaty Woodpecker (Baral and Huettmann 2020). The species exhibits the least habitat suitability in built‐up areas (Figure 6), underscoring its strong association with forested regions containing large trees and minimal human intervention.

While our study focused on climatic factors to understand the habitat distribution of the Great Slaty Woodpecker in Nepal, it is important to note that this rare species is confined to dense forests with mature trees and has also been observed at forest edges near human settlements. Our models did not account for other ecological factors, such as food availability, nest predation, or other microhabitat selection, which may also influence the species' habitat preferences. Future research should include these elements to better assess the population status and habitat distribution of the species, particularly in the context of increasing anthropogenic pressures. Addressing these gaps will be crucial for developing comprehensive conservation strategies to protect this vulnerable species and its habitat in Nepal and beyond.

## 5 Conservation Implications

The dependence of the Great Slaty Woodpecker on large trees and mature forest ecosystems makes it highly vulnerable to habitat degradation and loss, emphasizing the critical need to protect these habitats for its survival. Furthermore, species' significant habitat has been recorded outside the protected areas. To ensure the long‐term survival of the Great Slaty Woodpecker, conservation strategies must extend beyond protected areas to include community forests and other site‐based conservation measures. Protecting mature trees, restoring degraded habitats, and promoting sustainable forest management practices are essential for maintaining suitable habitats for this vulnerable species. Additionally, timely researches to investigate the species' nesting ecology, food availability, and responses to anthropogenic pressures will help to design and implement effective conservation planning.

## 6 Conclusion

This study provides the most comprehensive assessment to date of the species' population status and habitat distribution in Nepal. Our findings highlight the species' strong dependence on large trees and mature forests, as well as its vulnerability to habitat degradation and climatic factor association. Suitability of sites outside the protected area system and occurrences in disturbed habitats such as degraded forest edges and agricultural areas indicate increased vulnerability to survival. Conservation strategies must prioritize the protection of mature forests, both within and outside protected areas, to safeguard the remaining populations of the Great Slaty Woodpecker. By addressing these challenges, we can ensure the long‐term survival of this ecologically important species and the biodiversity it supports.

## Author Contributions

**Deelip Chand Thakuri:** conceptualization (lead), data curation (lead), formal analysis (equal), funding acquisition (lead), investigation (lead), methodology (equal), writing – original draft (lead). **Hari Basnet:** data curation (equal), formal analysis (equal), investigation (supporting), writing – review and editing (lead). **Laxman Prasad Poudyal:** resources (lead), supervision (supporting), writing – review and editing (supporting). **Prakash Chandra Aryal:** formal analysis (equal), methodology (equal), supervision (lead), writing – review and editing (equal).

## Conflicts of Interest

The authors declare no conflicts of interest.

## Data Availability

All the data regarding the manuscript can be found via Dryad https://doi.org/10.5061/dryad.fn2z34v4s.

## Appendix I Threshold and AUC of the Model

**Table:**

Replicates	0	1	2	3	4	5	6	7	8	9	Average
Threshold	0.06	0.1	0.1	0.05	0.11	0.14	0.02	0.08	0.04	0.1	0.08
AUC	0.979	0.979	0.96	0.974	0.945	0.958	0.977	0.957	0.978	0.969	0.9676
TSS	0.86	0.84	0.86	0.85	0.87	0.86	0.85	0.86	0.85	0.86	0.856

## Appendix II Status of Great Slaty Woodpecker at Different Habitats

**Table:**

Survey areas	Presence points	Total individuals
Overall	21	81
Inside protected areas	16	66
Outside protected areas	5	15
No logging	14	57
Logging	7	24
